# Gut Microbiota-Related Evidence Provides New Insights Into the Association Between Activating Transcription Factor 4 and Development of Salt-Induced Hypertension in Mice

**DOI:** 10.3389/fcell.2020.585995

**Published:** 2020-11-13

**Authors:** Tian-hao Liu, Wen-cong Tao, Qiu-er Liang, Wan-qing Tu, Ya Xiao, Li-guo Chen

**Affiliations:** ^1^College of Chinese Medicine, Jinan University, Guangzhou, China; ^2^Institute of Integrative Chinese and Western Medicine, Jinan University, Guangzhou, China

**Keywords:** hypertension, gene regulation, gut microbiota, vitamin K_2_, mice

## Abstract

Activating transcription factor 4 (ATF4), which regulates genes associated with endoplasmic reticulum stress, apoptosis, autophagy, the gut microbiome, and metabolism, has been implicated in many diseases. However, its mechanistic role in hypertension remains unclear. In the present study, we investigated its role in salt-sensitive hypertensive mice. Wild-type (WT) C57BL/6J mice were used to establish *Atf4* knockout (KO) and overexpression mice using CRISPR-Cas9 and lentiviral overexpression vectors. Then, fecal microbiota transplantation (FMT) from *Atf4*^±^ mice and vitamin K_2_ (VK2) supplementation were separately carried out in high-salt-diet (8% NaCl)-induced mice for 4 weeks. We found that *Atf4* KO inhibited and *Atf4* overexpression enhanced the increase in blood pressure and endothelial dysfunction induced by high salt intake in mice, while regulating the gut microbiota composition and VK2 expression. It was further verified that ATF4 is involved in the regulation of salt-sensitive hypertension and vascular endothelial function, which is achieved through association with gut microbiota and may be related to VK2 and different bacteria such as *Dubosiella*. In addition, we found that VK2 supplementation prevents the development of salt-sensitive hypertension and maintains vascular endothelial function; moreover, VK2 supplementation increases the abundance of intestinal *Dubosiella* and downregulates the relative expression of *Atf4* in the thoracic aorta of mice. We conclude that ATF4 plays an important role in regulating gut microbiota and VK2 production, providing new insights into the association between ATF4 and development of salt-induced hypertension in mice, meanwhile contributing to the development for a new preventive strategy of hypertension.

## Introduction

Hypertension is a systemic disease characterized by elevated arterial pressure and can be accompanied by damage to the heart, brain, kidney, blood vessels, and other target organs (Wang et al., 2018). According to a previous study, in China, the prevalence rate of hypertension in adults over 18 years of age is 23.2%, and the number of patients is 245 million; the prevalence rate of high blood pressure is 41.3%, and the number of patients is 435 million (Wang et al., 2018). Hypertension has become the main risk factor for the incidence of and death due to cardiovascular and cerebrovascular diseases such as stroke and coronary heart disease in China (Wang et al., 2018). Hypertension is a complex disease affected by both genetic and environmental factors, and salt intake is one of the important environmental factors. Individual blood pressure responses to salt load or salt limitation differ within the population, and the phenomenon called salt sensitivity is observed (Wilck et al., 2017; Paczula et al., 2019). Excessive salt intake leads to vascular endothelial dysfunction, which promotes and sustains the occurrence and development of hypertension—one of the pathogenesis of salt-sensitive hypertension (Wilck et al., 2017; Bier et al., 2018). Therefore, study of the occurrence and development of hypertension and effective prevention and treatment of hypertension constitute an important issue.

Activating transcription factor 4 is a member of the ATF/CREB transcription factor family and is associated with endoplasmic reticulum stress, apoptosis, and autophagy (Tameire et al., 2019; Xu et al., 2019; Wu et al., 2020). A previous study found that blocking the expression of ATF4 could promote cancer cells to produce much proteins and die (Tameire et al., 2019). A multi-group analysis of transcriptome, proteome, and metabolomics suggested that ATF4 was a key regulator of mitochondrial stress response in mammals, which provided a theoretical basis for the study of mitochondrial dysfunction and other related diseases (Quirós et al., 2017). Another study found that ATF4 directly regulated the expression of SLC1A5, which affected the levels of glutamine and expression of antimicrobial peptides in intestinal cells; thus, ATF4 may regulate the mechanism of IBD (Hu et al., 2019). In recent years, the research done at our research center has mainly focused on the relationship between vascular endothelial dysfunction and hypertension, including endothelial inflammatory response, endoplasmic reticulum stress, apoptosis, and miRNA mechanism. A study of cases of hypertension showed that ATF4 was the target of miR-1283—an miRNA associated with hypertension—and significant differences were observed between patients with hypertension and healthy volunteers (He et al., 2016a, b). However, the specific mechanism of action of ATF4 in the development of hypertension remains unclear. Therefore, we use a novel approach to investigate the association between ATF4 and hypertension and the mechanism of action of ATF4.

Previous studies have shown that gut microbiota disorders are closely associated with hypertension (Jing et al., 2017; Touyz and Camargo, 2019). Gut microbiota not only plays a role in regulating blood pressure but also regulates immune, neurological, and endocrine functions through their metabolites (Hendrik et al., 2019; Tang et al., 2019). For example, propionate can effectively reduce inflammation, atherosclerosis, and hypertensive cardiac remodeling and protect target organs (Hendrik et al., 2019). VK2—a gut microbial metabolite—suppresses inflammation, inhibits vascular smooth muscle cell apoptosis and prevents vascular calcification, and it is also associated with several cardiovascular diseases (Bhalerao and Clandinin, 2012; Vos et al., 2012; Ren et al., 2020). Bentley et al. illustrated the VK2 biosynthesis pathway related to bacteria in 1971 (Bentley and Meganathan, 1982; Ren et al., 2020). Ponziani et al. (2017) found gut microbiota were the main source of VK2 in humans, and small-intestinal bacterial overgrowth was associated with altered VK2 metabolism. Moreover, a higher intake of VK2 produced by gut microbiota was associated with lower risk of coronary heart disease (Haugsgjerd et al., 2020). Studies have shown that gut microbiota may interact with ATF4 through metabolites, and intestinal bacteria-related proteins are associated with ATF4 (Hodin et al., 2011; Sakai et al., 2019). Hu et al. (2019) found that ATF4 directly regulates the transcriptional activation of glutamine in intestinal epithelial cells, thus maintaining the function of Paneth cells secreting antimicrobial peptides, suggesting a role of ATF4 in maintaining the intestinal microenvironment. Therefore, it is of great importance to study the mechanisms of ATF4 involvement in the development of hypertension from the perspective of gut microbiota. On the basis of previous studies, we hypothesize that ATF4 regulates the balance of gut microbiota and mediates VK2 expression, which leads to endothelial dysfunction, thus participating in the development of hypertension.

**FIGURE 1:**
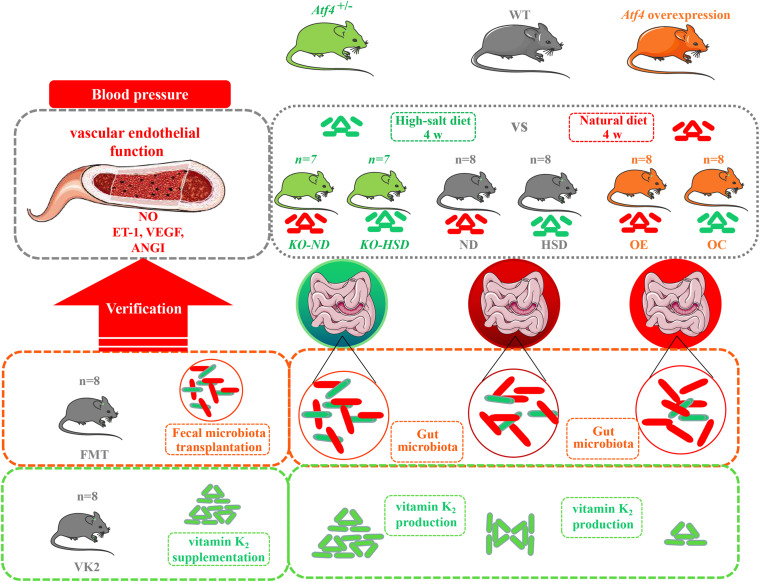
The cartoon of experimental design to investigate the potential mechanism of ATF4 participating in high-salt diet-induced hypertension. Wild-type (WT) C57BL/6J mice, *Atf4* knockout (*Atf4*^±^) and *Atf4* overexpression mice were used and high-salt-diet (8% NaCl) for 4 weeks was used to induce hypertensive mice. Then, fecal microbiota transplantation from *Atf4*^±^ mice and vitamin K_2_ supplementation were separately carried out to verify the gut microbiota-related evidence of the association between ATF4 and blood pressure. ATF4, activating transcription factor 4; KO, *Atf4*^±^ mice; WT, wild-type mice; NO, nitric oxide; VEGF, vascular endothelial growth factor; ANGI, angiotensin I; ET-1, endothelin-1; KO-ND, *Atf4*^±^ mice with natural diet; KO-HSD, *Atf4*^±^ mice with high-salt diet; ND, WT mice with natural diet; HSD, WT mice with high-salt diet; OE, *Atf4* overexpression with high-salt diet; OC, *Atf4* overexpression control with high-salt diet; FMT, fecal microbiota transplantation; VK2, vitamin K2.

In the present study, wild-type (WT) C57BL/6J mice were used to construct *Atf4* knockout (KO) (*Atf4*^±^) and *Atf4* overexpression mice using CRISPR-cas9 and lentiviral vectors. High-salt (8% NaCl)-induced mice were subjected to fecal microbiota transplantation (FMT) from *Atf4*^±^ mice and VK2 intervention. The cartoon of experimental design to investigate the potential mechanism of ATF4 participating in high-salt diet-induced hypertension is shown in Figure 1.

## Materials and Methods

### Animals and Study Design

All mice used in the study were of the C57BL/6J background. WT male mice (10–12-week-old) were purchased from the Experimental Animal Center of Guangzhou University of traditional Chinese Medicine. *Atf4* KO mice were established using theCas9/RNA system gene targeting technology by the Nanjing Biomedical Research Institute of Nanjing University (item no: T002330, Nanjing, China). Using the Cas9/RNA system gene targeting technology, sgRNA targeting ATF4 Exon2–3 gene was constructed, and Cas9 protein was directed to cut DNA double strand at specific sites of crRNA guided sequence target, thus resulting in sequence deletion. After microinjection of gRNA and cas9 into fertilized eggs of WT mice, F0 mice were produced after transplantation. The F1 generation mice were obtained by mating male and female of F0 generation mice and identified as positive. The F2 generation was obtained by mating male and female of F1 generation mice and identified as positive. Finally, male F2 heterozygous mice (10–12-week-old) were used for subsequent experiments. The use of the *Atf4*^±^ heterozygous mice was based on the fact that *Atf4*^–/–^ null mice have been also found to be mostly neonatal lethal, and even surviving mice are dwarf and immunocompromised (Masuoka and Townes, 2002; Wang et al., 2009; Cornejo et al., 2013). All mice were housed at the Experimental Animal Center of Jinan University in a pathogen-free environment under 12 h dark and 12 h light conditions. After being housed for 10 days to allow for adaptation, the KO mice were randomly grouped into two groups: *Atf4*^±^ mice with natural diet (KO-ND) and *Atf4*^±^ mice with high-salt diet (KO-HSD). The WT mice were randomly divided into the following groups: natural diet (ND), high-salt diet (HSD), *Atf4* overexpression with high-salt diet (OE), *Atf4* overexpression control with high-salt diet (OC), FMT, and VK2.

### Establishment of *Atf4* Overexpression Mice by Lentiviral Injection

Recombinant lentivirus containing *Atf4* was purchased from GeneChem (production license: GOSL0206520, Shanghai, China). WT mice in the OE group were infected with lentivirus containing *Atf4* (1 × 10^6^ infectious units per mice) by injection through the tail vein, whereas WT mice in the OC group were infected with the lentivirus (LVCON238) which provided by GeneChem (production license: 1341609, Shanghai, China) by equivalent injection into the tail vein to act as the negative control (Huang et al., 2014).

### Hypertension Induced by High-Salt Diet in Mice

The mouse model of hypertension was induced by a high-salt (8% NaCl) diet for 4 weeks (Zhang et al., 2018). The 8% high-salt feed was purchased from Teluofei (production license: [2014] 06092, Nantong, China) and sterilized by Nantong Michael Irradiation Co., Ltd. (sterilization batch: 19072705, Nantong, China).

### Fecal Microbiota Transplantation

Fresh feces (0.5 g) were collected from *Atf4*^±^ mice fixed to the operating table and were placed into a 50 mL centrifuge tube. After weighing the collected feces, normal saline was added in the ratio of 1:10 (Liu et al., 2017; Yan et al., 2018). After sufficient mixing, the fecal samples were centrifuged at 3000 r/min for 5 min. The fecal bacterial suspension was transferred to a sterile centrifuge tube with a pipette and then was administered by gavage to mice in the FMT group, within 2 h of preparation (1 mL/day per mice) (Liu et al., 2017; Yan et al., 2018).

### VK2 Diet

The mice in the VK2 group were fed a high-salt diet containing 0.036% VK2 (100 mg/kg/day), which was provided by Nantong Teluofei Feed Technology Co., Ltd. (production license: [2014] 06092, Nantong, China) and sterilized by Nantong Michael Irradiation Co., Ltd. (sterilization batch: 19072705). VK2 was purchased from Sanitary (China) Pharmaceutical Co., Ltd. (registration certificate: h20100462).

### Systolic Blood Pressure Measurements

Systolic blood pressure of the caudal artery was measured manometrically using a blood pressure measurement system. When the baseline body temperature of all mice to be tested was stable at 37°C, the mice were fixed on the test platform. The measurements were taken when the waveform was stable and the measurement results were constant. The mice were placed in the mouse fixator on the test platform to warm up for approximately 3 min to allow them to adapt to the environment of the test platform, and then the tail of the mice was placed through the sensor at the bottom of the V-groove. The blood pressure analysis program (Visitech Systems, BP-2000-M) was used for the measurements. Measurements were performed 15 times per mouse. The average value was recorded and measured at 3 p.m. The blood pressure measurements were taken once a week, and all measurements were taken by the same operator.

### Evaluation of Serum Levels of Endothelial Function-Related Factors by Enzyme-Linked Immunosorbent Assay (ELISA)

After 4 weeks of feeding, the mice were placed under sodium pentobarbital anesthesia to minimize suffering. Blood was collected from the eyeballs of the mice in a 5 mL blood collection tube. After 2–4 h, blood samples were centrifuged at 3000 r/min for 5 min, and the serum was collected in a sterilized centrifuge tube. The serum levels of NO, ET-1, ANGI, and VEGF were measured according to the instructions provided with the respective kits. The NO ELISA kit (production license: MM-0658M1/96T), ET-1 ELISA kit (production license: MM-0561M1/96), ANGI ELISA kit (production license: MM-0128M1/96T), and VEGF ELISA kit (production license: MM-0397M1/96T) were purchased from MEIMIAN (Yancheng, China).

### Examination of Endothelial Morphology by Transmission Electronic Microscope (TEM)

After being fixed in 2.5% glutaraldehyde at 4°C for 2–4 h to minimize the mechanical injury such as traction, contusion, and extrusion, the mouse aortic tissue was fixed and rinsed three times using 0.1 M phosphate buffer Pb (pH7.4). After dehydration and infiltration, the tissue of mice was cut into 60–80 nm ultrathin sections. Then these sections were stained with uranium and lead (2% uranium acetate saturated alcohol solution, lead citrate), each for 15 min. Finally, three images were collected and analyzed from each group under a transmission electron microscope (HT7700; Hitachi; Tokyo, Japan).

### Genomic DNA Extraction, 16S rRNA Gene Sequencing, and Gut Microbiota Composition Analysis

Genomic DNA was extracted from the fecal samples of mice (six mice randomly selected from each group) using the E.Z.N.A.^®^ soil DNA Kit (Omega Bio-tek, Norcross, GA, United States) according to manufacturer’s protocol. The DNA concentration and purity were determined by NanoDrop 2000 UV–vis spectrophotometer (Thermo Scientific, Wilmington, DE, United States), and DNA quality was checked using 1% agarose gel electrophoresis. The V3–V4 hypervariable regions of the bacterial 16S rRNA gene were amplified with primers 338F (5′-ACTCCTACGGGAGGCAGCAG-3′) and 806R (5′-GGACTACHVGGGTWTCTAAT-3′) using a thermocycler PCR system (GeneAmp 9700, ABI, Carlsbad, CA, United States). PCR was conducted using the following program: 3 min of denaturation at 95°C; 27 cycles of 30 s at 95°C, 30 s for annealing at 55°C, and 45 s for elongation at 72°C; and a final extension for 10 min at 72°C. The PCR reactions were performed in triplicate. The reaction mixture (20 μL) contained 4 μL of 5x FastPfu buffer, 2 μL of 2.5 mM dNTPs, 0.8 μL of each primer (5 μM), 0.4 μL of FastPfu polymerase, and 10 ng of template DNA. The resulting PCR products were extracted from a 2% agarose gel and further purified using the AxyPrep DNA Gel Extraction Kit (Axygen Biosciences, Union City, CA, United States) and quantified using QuantiFluor^TM^-ST (Promega, Madison, WI, United States) according to the manufacturer’s protocol.

Purified amplicons were pooled in equimolar quantities and subjected to paired-end sequencing (2 × 300) on an Illumina MiSeq platform (Illumina, San Diego, CA, United States), according to the standard protocols, by Majorbio Bio-Pharm Technology Co. Ltd. (Shanghai, China). OTUs were clustered with 97% similarity cutoff using UPARSE (version 7.1^1^) with a novel “greedy” algorithm that performs chimera filtering and OTU clustering simultaneously. The taxonomy of each 16S rRNA gene sequence was analyzed by RDP Classifier algorithm^2^ against the Silva (SSU123) 16S rRNA database using confidence threshold of 70%. Principal component analysis (PCA) and non-metric multidimensional scaling (NMDS) were performed using R package (MathSoft, Inc., United States) to display the main distribution characteristics and similarity of samples. The data of 16S rRNA gene sequencing were analyzed using the free online Majorbio I-Sanger Cloud Platform^3^.

### Evaluation of VK2 by ELISA

Serum VK2 was quantified using a VK2 ELISA kit provided by MEIMIAN (production license: MM-44662M1/96T, Yancheng, China) according to manufacturer’s instructions.

### Expression of ATF4 in Aortic Tissue

#### Real-Time PCR

RNA from the aortic and intestinal tissues of mice (three mice randomly selected from each group) were isolated using TRIzol (Invitrogen), and reverse transcription was carried out using the first-strand cDNA synthesis kit (TaKaRa). Real-time PCR (RT-PCR) was performed using EvaGreen dye (Biotium) in an ABI PRISM^®^ 7500 sequence detection system. Primer sequences are summarized in Supplementary Table 1. The relative expression level of ATF4 mRNA was calculated using the 2^–ΔΔ*CT*^ method with glyceraldehyde-3-phosphate dehydrogenase (GAPDH) as an internal reference.

#### Western Blotting

Total protein was extracted from the aortic and intestinal tissue of mice (three mice randomly selected from each group). Phenylmethylsulfonyl fluoride (10 μL; 100 mM) and cocktail (10 μL) were added to 1 mL cracking solution. The protein concentration was determined using the BCA protein assay kit (KGPBCA, KeyGenBiotech). Equal quantities of protein were subjected to western blot analysis. Then, the membranes were washed with Tris-HCl-Tween buffer salt solution (TBST), and the signal was enhanced by chemiluminescence. The expression of ATF4 protein was observed using the gel imager. ATF4 Monoclonal Antibody (CST 11815S), GAPDH Loading Control Antibody (CST 51332S), and Goat Anti-Mouse IgG Antibody (CST 14709S) were used. ATF4 protein expression is presented as the ratio of ATF4 protein/GAPDH.

### Statistical Analysis

All data are presented as mean ± standard deviation (SD). Statistical analysis was performed using GraphPad Prism 5 (GraphPad Software Inc., United States). Statistical comparisons were performed using Student *t*-test or one-way analysis of variance (ANOVA). *p* < 0.05 was considered as statistically significant.

## Results

### ATF4 Altered Systolic Blood Pressure and Endothelial Function in High-Salt-Induced Mice

To validate and verify ATF4 expression, we investigated the protein and mRNA levels of ATF4 in mouse aortic and intestinal tissues using western blotting and RT-PCR, respectively. As shown in Supplementary Figure 1, ATF4 protein expression in *Atf4*^±^ mice was indeed lower than that in WT mice (Supplementary Figures 1A,B,D,E). A high-salt diet significantly increased the ATF4 protein expression in WT mice, and overexpression of *Atf4* also significantly increased the ATF4 protein expression in WT mice (Supplementary Figures 1A,B,D,E). Moreover, ATF4 mRNA expression in *Atf4*^±^ mice was lower than that in WT mice (Supplementary Figures 1C,F). A high-salt diet significantly increased the expression of ATF4 mRNA in WT mice. Overexpression of *Atf4* also significantly increased the expression of ATF4 mRNA in WT mice (Supplementary Figures 1C,F). These data suggested that ATF4 expression in *Atf4*^±^ mice was suitable for use in the current study and ATF4 plays a pivotal role in the pathogenesis of high-salt diet in mice.

**FIGURE 2:**
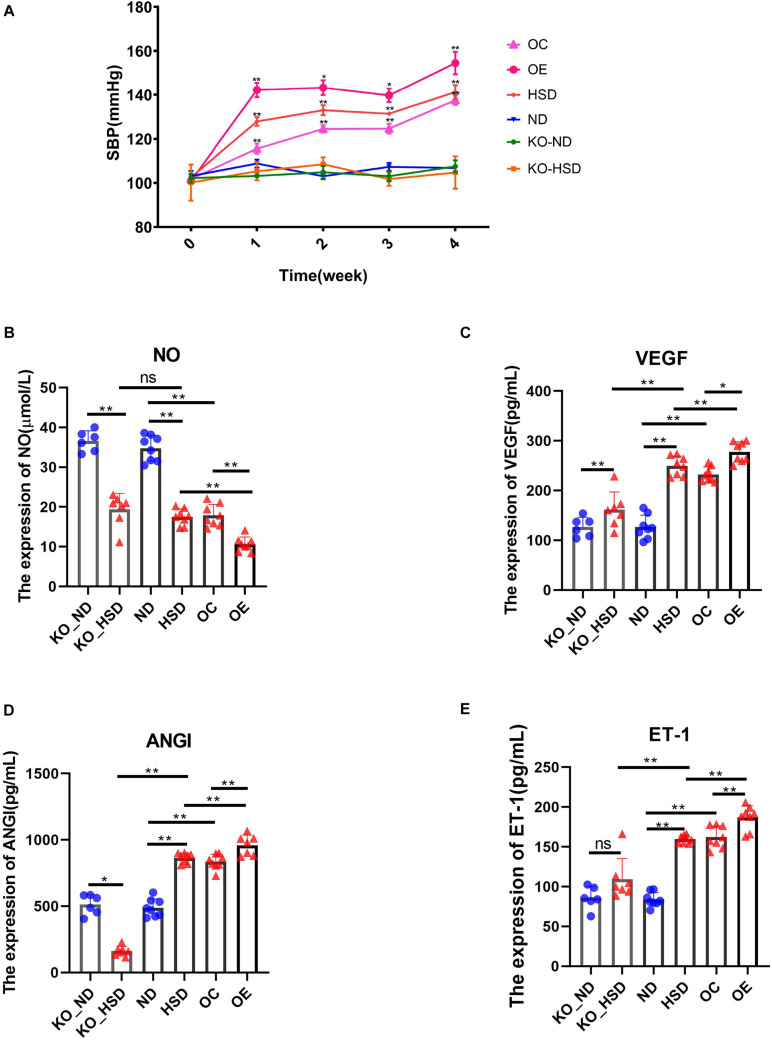
ATF4 altered SBP and endothelial function-related factors in high-salt-induced hypertensive mice. **(A)** ATF4 altered SBP in high-salt-induced hypertensive mice. ND vs HSD, HSD vs OE, OE vs OC, *p < 0.05 and **p < 0.01. **(B)** ATF4 altered the expression of NO in high-salt-induced hypertensive mice. **(C)** ATF4 altered the expression of VEGF in high-salt-induced hypertensive mice. **(D)** ATF4 altered the expression of ANGI in high-salt-induced hypertensive mice. **(E)** ATF4 altered the expression of ET-1 in high-salt-induced hypertensive mice. Data are presented as mean ± SD; ns, no significance, *p < 0.05 and **p < 0.01, *n* = 6–8; statistical comparisons were performed using Student *t*-test or one-way analysis of variance (ANOVA). ATF4, activating transcription factor 4; KO, *Atf4*^±^ mice; WT, wild-type mice; SBP, systolic blood pressure; NO, nitric oxide; VEGF, vascular endothelial growth factor; ANGI, angiotensin I; ET-1, endothelin-1; KO-ND, *Atf4*^±^ mice with natural diet; KO-HSD, *Atf4*^±^ mice with high-salt diet; ND, WT mice with natural diet; HSD, WT mice with high-salt diet; OE, *Atf4* overexpression with high-salt diet; OC, *Atf4* overexpression control with high-salt diet.

In order to investigate the association between ATF4 and blood pressure, we observed the variations in blood pressure in mice for 4 weeks. It was found that the blood pressure of WT mice increased significantly after 4 weeks of high-salt diet; the blood pressure increased to a higher degree in *Atf4* overexpression mice but did not increase in *Atf4*^±^ mice, indicating that ATF4 contributes to the development of hypertension induced by high salt intake in mice (Figure 2A). A previous study showed that mechanisms of salt-sensitive hypertension were primarily derived from vascular dysfunction and characterized by endothelial dysfunction (Kurtz et al., 2016). To investigate whether the increase in blood pressure induced by a high-salt diet in mice was linked to endothelial dysfunction, we examined the endothelial function by analyzing the serum levels of NO, VEGF, ANGI, and ET-1. High-salt diet significantly reduced the serum levels of NO in WT mice; *Atf4* overexpression also significantly lowered the serum levels of NO (Figure 2B). High-salt diet significantly increased the serum levels of VEGF, ANGI, and ET-1 in WT mice; after overexpression of *Atf4*, high-salt diet also significantly increased higher (Figures 2C–E). The serum levels of VEGF and ET-1 in the *Atf4*^±^ mice induced by high-salt diet were less than that in WT mice induced by high-salt diet, whereas the serum levels of NO in the *Atf4*^±^ and WT mice induced by high-salt diet were not significant (Figures 2B–E). In addition, significantly decreased serum levels of ANGI were found in the *Atf4*^±^ mice induced by high-salt diet and the serum levels of ANGI were less than that in WT mice induced by high-salt diet (Figure 2D).

### ATF4 Altered the Gut Microbiota Composition and VK2 Levels in High-Salt-Induced Hypertensive Mice

**FIGURE 3:**
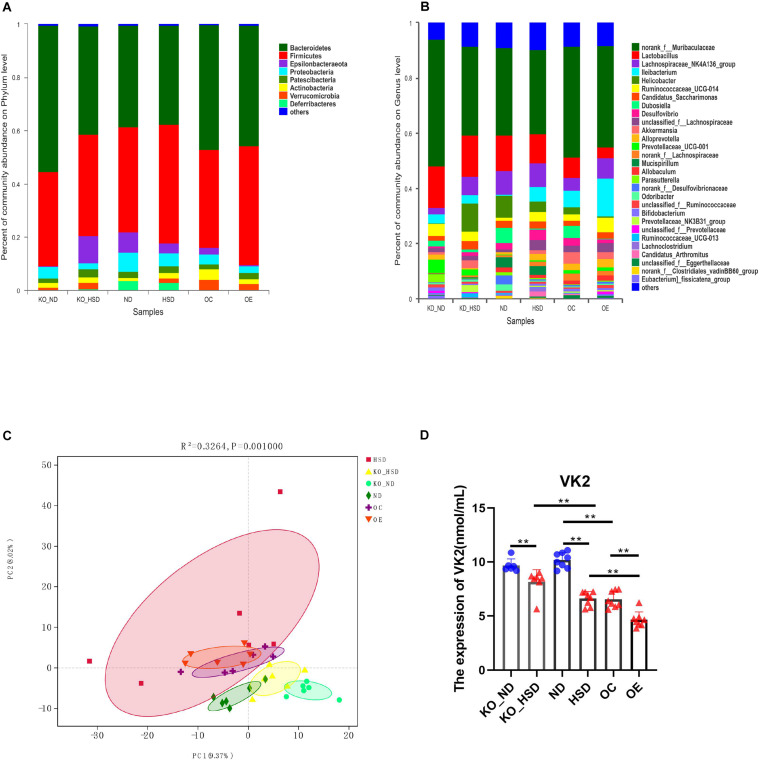
ATF4 altered the composition of gut microbiota and expression levels of the microbial metabolite VK2 in high-salt-induced hypertensive mice. **(A)** Relative abundance plots displaying the differences in the microbial community structure at the phylum level. **(B)** Relative abundance plots displaying the differences in the general microbial community structure. **(C)** PCA plots were used to visualize differences in weighted UniFrac distances of samples of OTUs from different groups (*R*^2^ = 0.3264, *p* = 0.001). **(D)** ATF4 altered the expression of VK2. Data are presented as mean ± SD; **p < 0.01, *n* = 6–8; statistical comparisons were performed using Student t test or one-way analysis of variance (ANOVA). ATF4, activating transcription factor 4; KO, *Atf4*^±^ mice; WT, wild-type mice; SBP, systolic blood pressure; NO, nitric oxide; VEGF, vascular endothelial growth factor; ANGI, angiotensin I; ET-1, endothelin-1; KO-ND, *Atf4*^±^ mice with natural diet; KO-HSD, *Atf4*^±^ mice with high-salt diet; ND, WT mice with natural diet; HSD, WT mice with high-salt diet; OE, *Atf4* overexpression with high-salt diet; OC, *Atf4* overexpression control with high-salt diet.

To exhibit the mechanisms of action of ATF4 in the development of hypertension induced by high salt in mice, we analyzed composition of the gut microbiota and serum levels of the microbial metabolite VK2 in high-salt-induced hypersensitive mice. As shown in Figures 3A,B, the species of dominant phyla and genera of ATF4 participation in the development of hypertension induced by high salt in mice did not change, but the relative abundance of dominant phyla and genera changed. The PCA plot revealed significant differences in gut microbial community structure of ATF4 participation in the development of hypertension induced by high salt in mice (*R*^2^ = 0.3264, *p* = 0.001; Figure 3C). Using ELISA, we examined whether the serum levels of the microbial metabolite VK2 differed among groups. It was found that a high-salt diet significantly reduced the expression of VK2 in WT and *Atf4*^±^ mice after 4 weeks. Moreover, a high-salt diet significantly reduced the expression of VK2 in the *Atf4* overexpression mice. In addition, the decrease of VK2 in the WT mice induced by high salt intake was lower than that in the *Atf4*^±^ mice (Figure 3D). These results suggest that ATF4 is involved in the regulation of gut microbiota in high-salt-induced hypertensive mice, and the regulation of blood pressure by ATF4 may be associated with gut microbiota and VK2.

### Microbial Correlation

**FIGURE 4:**
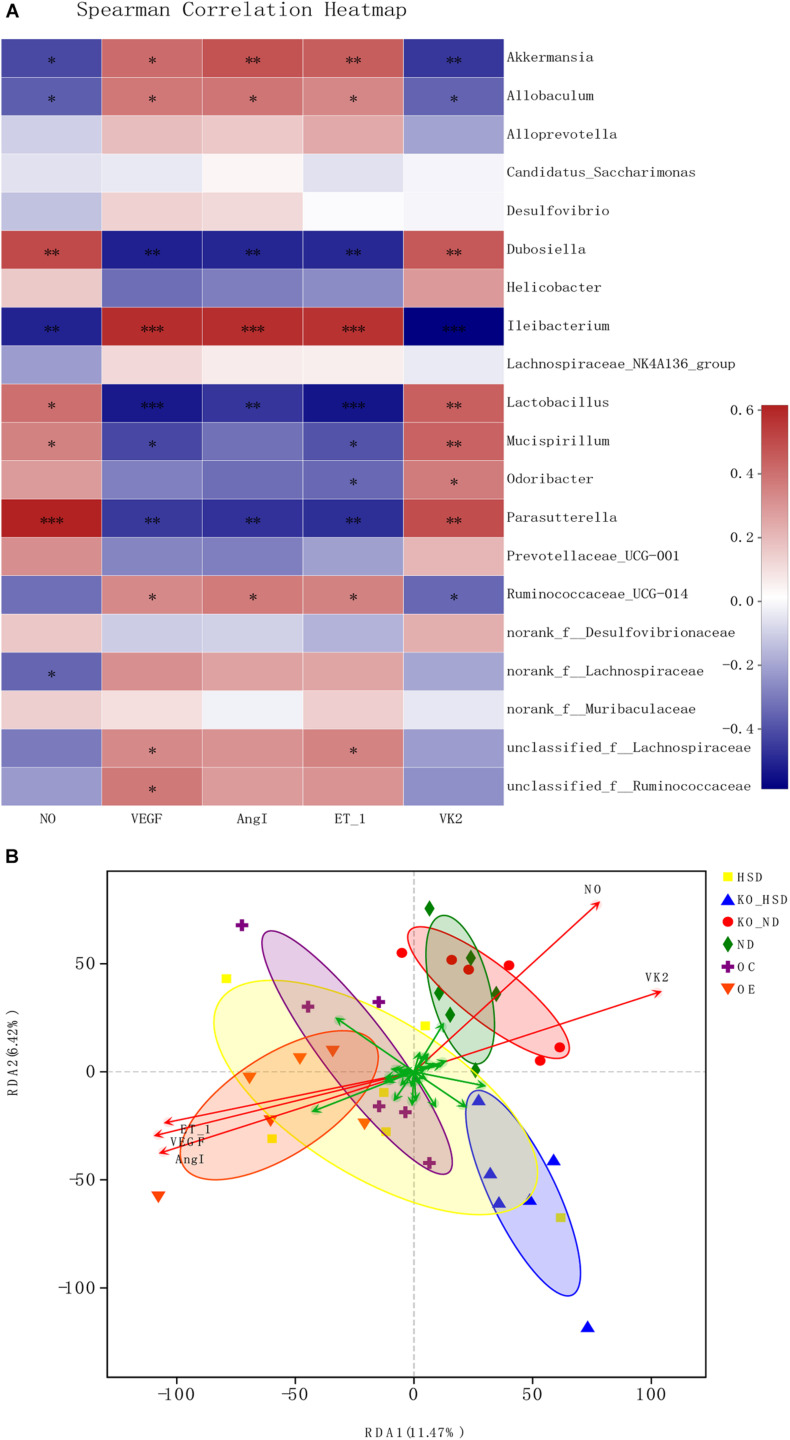
Microbial correlation in high-salt-induced hypertensive mice. **(A)** Spearman correlation analysis between top 20 genera and environmental factors. Red represents positive correlation, whereas blue represents negative correlation; the darker the color, the stronger the correlation. ^∗^0.01 < *p* ≤ 0.05, ^∗∗^0.001 < *p* ≤ 0.01, ^∗∗∗^*p* ≤ 0.001. **(B)** RDA analysis of environmental factors. The angle between the arrows of different environmental factors represents positive and negative correlations (acute angle: positive correlation; obtuse angle: negative correlation; right angle: no correlation). ATF4, activating transcription factor 4; KO, *Atf4*^±^ mice; WT, wild-type mice; SBP, systolic blood pressure; NO, nitric oxide; VEGF, vascular endothelial growth factor; ANGI, angiotensin I; ET-1, endothelin-1; KO-ND, *Atf4*^±^ mice with natural diet; KO-HSD, *Atf4*^±^ mice with high-salt diet; ND, WT mice with natural diet; HSD, WT mice with high-salt diet; OE, *Atf4* overexpression with high-salt diet; OC, *Atf4* overexpression control with high-salt diet.

Spearman correlation analysis of the factors associated with vascular endothelial function and gut microbiota as well as VK2 indicated that the top 20 genera, such as *Akkermansia*, *Allobaculum*, *Dubosiella*, *Ileibacterium*, *Lactobacillus*, *Mucispirillum*, *Parasutterella*, and *Ruminococcaceae_UCG-014*, showed a positive or negative correlation with levels of NO, VEGF, ANGI, ET-1, and VK2 (Figure 4A). Additionally, RDA correlation analysis showed a positive correlation between VK2 and NO levels and a negative correlation between VK2 levels and VEGF, ANGI, and ET-1 levels (Figure 4B).

### Fecal Microbiota Transplantation From *Atf4*^±^ Mice Improved Systolic Blood Pressure and Altered Endothelial Function-Related Factor Expression in High-Salt-Induced Hypertensive Mice

**FIGURE 5:**
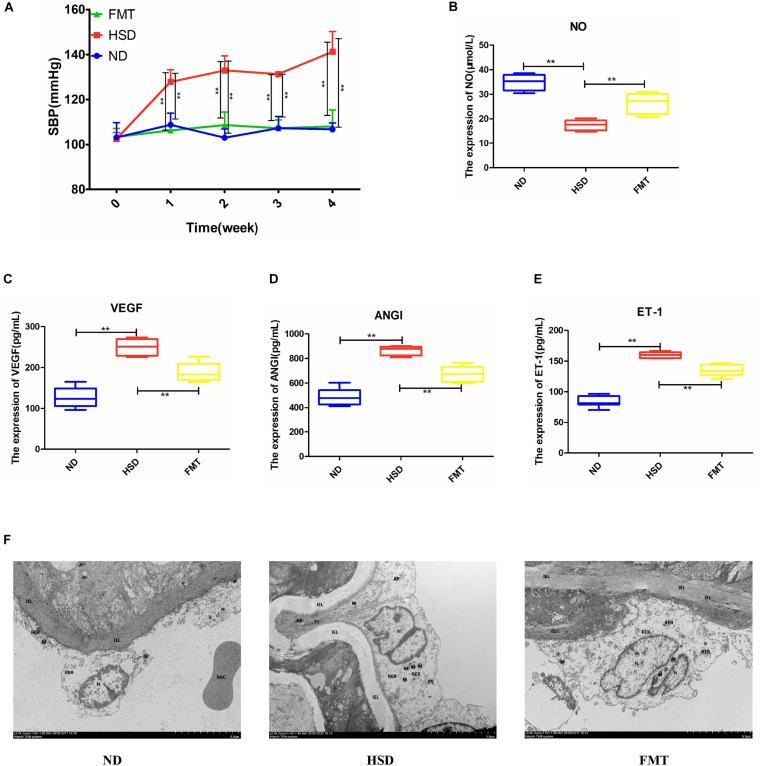
FMT using *Atf4*^±^ mice fecal microbiota improved SBP and altered expression of endothelial function-related factors in high-salt-induced hypertensive mice. **(A)** FMT maintained SBP. **(B)** FMT upregulated the expression of NO. **(C)** FMT downregulated the expression of VEGF. **(D)** FMT downregulated the expression of ANGI. **(E)** FMT downregulated the expression of ET-1. **(F)** Examination of endothelial morphology by transmission electronic microscope (TEM). IEL, internal elastic lamina; N, nucleus; M, mitochondrion; RER, rough endoplasmic reticulum; TJ, tight junction; AP, autophagy. Data are presented as mean ± SD; **p < 0.01, *n* = 6–8; statistical comparisons were performed using Student *t*-test or one-way analysis of variance (ANOVA). FMT, fecal microbiota transplantation; SBP, systolic blood pressure; NO, nitric oxide; VEGF, vascular endothelial growth factor; ANGI, angiotensin I; ET-1, endothelin-1; ND, WT mice with natural diet; HSD, WT mice with high-salt diet.

To verify whether involvement of ATF4 in BP and endothelial function is dependent on the regulation of gut microbiota in high-salt-induced hypertensive mice, we transplanted the fecal microbiota of *Atf4*^±^ mice into WT mice fed with a high-salt diet. The results showed that blood pressure of the WT mice induced by the high-salt diet increased over time, whereas blood pressure of the fecal microbiota transplanted mice did not significantly increase (Figure 5A). The serum levels of NO in the WT mice induced by the high-salt diet significantly decreased after 4 weeks and significantly increased after FMT (Figure 5B). In addition, the serum levels of VEGF, ANGI, and ET-1 in the WT mice induced by the high-salt diet significantly increased after 4 weeks and significantly decreased after FMT (Figures 5C–E). Moreover, the results of ultrastructural changes by TEM were shown in Figure 5F. Compared with the ND group, the results showed that the vascular endothelial cells in HSD had obvious edema, the intracellular matrix was obviously lightened, the electron density of large area was decreased, and the internal elastic lamina was partially broken; the rough endoplasmic reticulum was obviously expanded and degranulated; the nucleus showed local depression; the number of mitochondrion was small and swollen, and the mitochondrial cristae became shorter and disappeared; the intercellular space was significantly widened; there were tight junctions and autophagy. After FMT, the cell membrane of vascular endothelial cells was relatively intact; the nucleus was irregular and heterochromatin was edge gathered; the mitochondrion and the internal elastic lamina were not obviously broken (Figure 5F). All these findings suggested that the involvement of ATF4 in the regulation of blood pressure and endothelial function is a result of the gut microbiota in salt-sensitive hypertensive mice.

### Fecal Microbiota Transplantation From *Atf4*^±^ Mice Altered the Gut Microbiota Composition and VK2 Levels in High-Salt-Induced Hypertensive Mice

**FIGURE 6:**
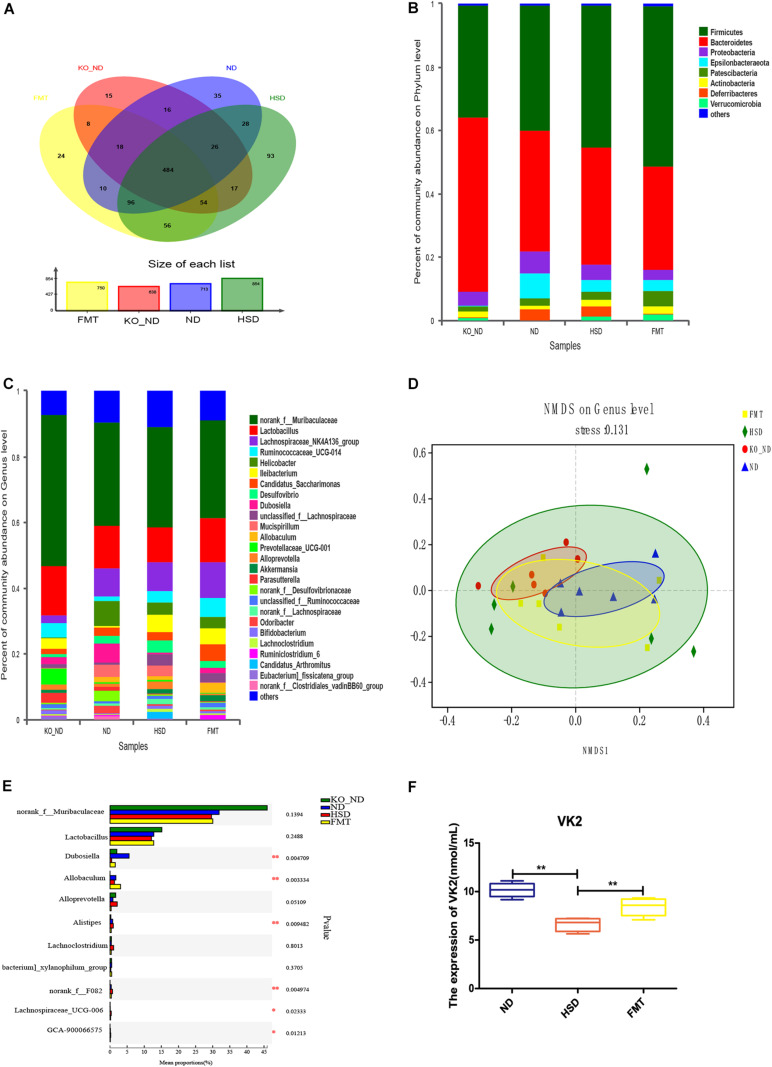
FMT using *Atf4*^±^ mice fecal microbiota altered the composition of gut microbiota and expression of the microbial metabolite VK2 in high-salt-induced hypertensive mice. **(A)** Venn diagram of OTUs. Different colors represent different groups. **(B)** Relative abundance plots displaying the differences in the microbial community structure at the phylum level. **(C)** Relative abundance plots displaying the differences in the general microbial community structure. **(D)** NMDS plots were used to visualize differences in weighted UniFrac distances at the genus level of samples from different groups. **(E)** The 11 taxonomic groups associated with FMT. *p < 0.05 and **p < 0.01. **(F)** FMT upregulated the expression of VK2. Data are presented as mean ± SD; **p < 0.01, *n* = 6–8; statistical comparisons were performed using Student *t*-test or one-way analysis of variance (ANOVA). FMT, fecal microbiota transplantation; VK2, vitamin K_2_; KO-ND, *Atf4*^±^ mice with natural diet; ND, WT mice with natural diet; HSD, WT mice with high-salt diet.

To ensure successful transplantation, a gut microbiota analysis was performed by 16S rRNA gene sequencing. The results showed that there were 638 OTUs in the feces of *Atf4*^±^ mice, 713 OTUs in the WT mice, 854 OTUs in the high-salt-diet-induced WT mice, and 750 OTUs in mice in the FMT group (Figure 6A). Thus, the gut microbiota composition differed before and after transplantation (Figures 6B–D), which could be significantly distinguished at the genus level (Figure 6D). Furthermore, we found 11 different genera based on the higher relative abundance and greater difference; the relative abundance of six of these differed significantly among groups (Figure 6E and Table 1; *p* < 0.05). The findings suggested that FMT from *Atf4*^±^ mice could decrease the relative abundance of *norank_f__Muribaculaceae*, *Lactobacillu*s, *Dubosiella*, *Alloprevotella*, *Allobaculum*, and [*Eubacterium]_xylanophilum_group* and increase the relative abundance of *Lachnoclostridium*, *norank_f__F082*, *Lachnospiraceae_UCG-006* and *GCA-900066575* in high-salt-induced hypertensive mice. Additionally, *Dubosiella* was selected according to the higher relative abundance, the higher fold change and the *p*-value < 0.05 at the same time, which suggest that *Dubosiella* may be associated with the development of salt-sensitive hypertension in which ATF4 is involved. Moreover, FMT significantly upregulated the serum levels of VK2 in the WT mice induced by the high-salt diet after 4 weeks (Figure 6F), indicating that the changes in VK2 resulted from the observed differences in the microbiota associated with ATF4.

**TABLE 1:** Relative abundance of fecal microbiota transplantation-related taxonomic groups (Mean ± SD; *n* = 6).

Name	KO_ND (%)	ND (%)	HSD (%)	FMT (%)	*p* value	Corrected *p*-value
*norank_f__Muribaculaceae*	45.93 ± 10.81	31.86 ± 12.8	29.63 ± 17.38	30.03 ± 9.48	0.1394	0.2993
*Lactobacillus*	15.14 ± 11.82	12.79 ± 6.729	12.1 ± 23.25	12.76 ± 10.61	0.2488	0.4555
*Dubosiella*	1.985 ± 0.5682	5.578 ± 2.641	0.4698 ± 0.4571	1.533 ± 0.6933	0.004709	0.03755
*Allobaculum*	0.005346 ± 0.005733	1.726 ± 1.028	1.292 ± 1.076	3.058 ± 2.642	0.003334	0.03396
*Alloprevotella*	1.672 ± 0.6219	0.8547 ± 0.3379	2.112 ± 2.561	0.327 ± 0.2928	0.05109	0.157
*Alistipes*	0.2481 ± 0.1307	0.7732 ± 0.2805	0.9667 ± 0.656	0.351 ± 0.1823	0.009482	0.06099
*Lachnoclostridium*	0.4284 ± 0.3213	0.3939 ± 0.1745	1.058 ± 1.364	0.3555 ± 0.2004	0.8013	0.8143
*[Eubacterium]_xylanophilum_group*	0.4528 ± 0.2188	0.4992 ± 0.3265	0.2361 ± 0.3438	0.4957 ± 0.496	0.3705	0.466
*norank_f__F082*	0.005244 ± 0.008202	0.3672 ± 0.2363	0.7269 ± 1.189	0.398 ± 0.322	0.004974	0.03755
*Lachnospiraceae_UCG-006*	0.027 ± 0.02584	0.06006 ± 0.03032	0.364 ± 0.3993	0.1974 ± 0.2518	0.02333	0.1019
*GCA-900066575*	0.006198 ± 0.004577	0.1095 ± 0.2425	0.2101 ± 0.3947	0.1041 ± 0.07371	0.01213	0.06937

*KO-ND, *Atf4*^±^ mice with natural diet; ND, WT mice with natural diet; HSD, WT mice with high-salt diet; FMT, fecal microbiota transplantation.*

### VK2 Maintained Systolic Blood Pressure and Endothelial Function-Related Factors in High-Salt-Induced Hypertensive Mice

**FIGURE 7:**
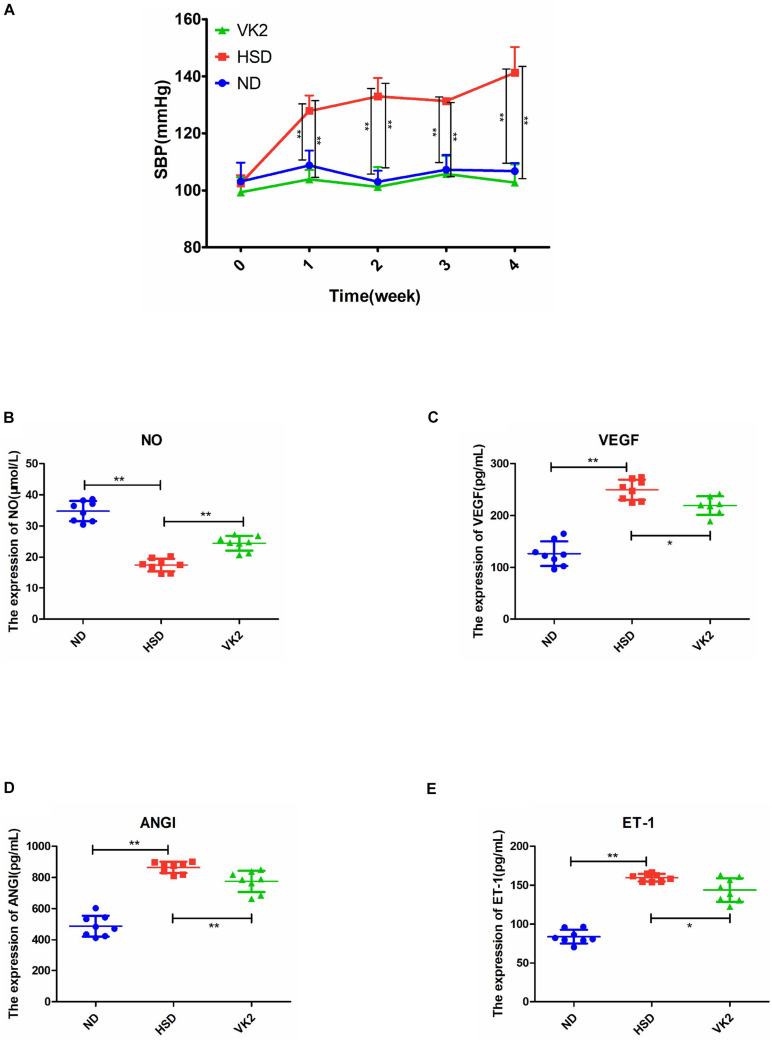
VK2 maintained SBP and altered endothelial function-related factors in high-salt-induced hypertensive mice. **(A)** VK2 altered SBP. **(B)** VK2 upregulated the expression of NO. **(C)** VK2 downregulated the expression of VEGF. **(D)** VK2 downregulated the expression of ANGI. **(E)** VK2 downregulated the expression of ET-1. Data are presented as mean ± SD; *p < 0.05 and **p < 0.01, *n* = 6–8; statistical comparisons were performed using Student *t*-test or one-way analysis of variance (ANOVA). SBP, systolic blood pressure; VK2, vitamin K_2_; NO, nitric oxide; VEGF, vascular endothelial growth factor; ANGI, angiotensin I; ET-1, endothelin-1; ND, WT mice with natural diet; HSD, WT mice with high-salt diet.

To assess whether VK2 was a target metabolite that may be associated with the regulation of blood pressure and endothelial function, VK2 was added into the high-salt diet to induce the mice. The results confirmed that VK2 supplementation could significantly maintain blood pressure in high-salt-induced mice (Figure 7A). Moreover, VK2 supplementation significantly increased the serum levels of NO in the high-salt-induced hypertensive mice (Figure 7B) and significantly decreased the serum levels of VEGF, ANGI, and ET-1 in hypertensive mice induced by the high-salt diet after 4 weeks (Figures 7C–E). Further analysis of serum VK2 confirmed that higher VK2 levels could be detected in the serum after high-salt diet containing VK2 was fed to mice for 4 weeks (Supplementary Figure 2). Furthermore, VK2 supplementation significantly increased the abundance of *Dubosiella*, which may be associated with the alterations in blood pressure and endothelial function due to ATF4 (Supplementary Figure 2). In addition, VK2 supplementation significantly decreased ATF4 expression (protein and mRNA levels) in high-salt-induced hypertensive mice (Supplementary Figure 3).

## Discussion

In the present study, we investigated the gene *Atf4* on the basis of previous studies and found it to be associated with hypertension (He et al., 2016a,b). Research showed vascular endothelial dysfunction could promote and sustain the occurrence and development of hypertension (Wilck et al., 2017; Bier et al., 2018). A previous study showed that mechanisms of salt-sensitive hypertension were primarily derived from vascular dysfunction and characterized by endothelial dysfunction (Kurtz et al., 2016). Studies have shown that NO, VEGF, ANGI, and ET-1 were related to vascular endothelial function (Xu et al., 2004; Rufaihah et al., 2017; Binder et al., 2020). To investigate whether the increase in blood pressure induced by a high-salt diet in mice was linked to endothelial dysfunction, we examined the endothelial function by analyzing the serum levels of NO, VEGF, ANGI, and ET-1. Moreover, gut microbiota could play a role in regulating blood pressure (Hendrik et al., 2019; Tang et al., 2019). Previous studies have not reported whether ATF4 is associated with endothelial function and blood pressure. Therefore, we sought to use *Atf4*^±^ and WT mice to determine whether ATF4 regulates the blood pressure and endothelial function in high-salt diet-induced mice and to investigate the microbiota-related mechanism of action of ATF4 in hypertension. Our major findings are as follows: First, ATF4 was involved in the development of salt-sensitive hypertension accompanied by changes in blood pressure and endothelial function in mice. Second, the gut microbiota was the associated intermediary between ATF4 and salt-sensitive hypertension, indicating that ATF4-related changes in blood pressure and endothelial function are due to gut microbiota. Third, the microbial metabolite VK2 had beneficial effects on salt-sensitive hypertension, which may be a microbiota-related molecular mechanism.

Recent studies have suggested that ATF4 is a target for the prevention and treatment of a variety of diseases. For example, it is believed that inhibitors of ATF4 protein synthesis may help to find new approaches to prevent cancer and that ATF4 may become a novel potential target for the treatment of IBD, mitochondrial dysfunction, and other related diseases (Quirós et al., 2017; Hu et al., 2019). Moreover, the studies revealed the relationship between gene and blood pressure regulation. For example, G-protein-coupled estrogen receptor 1 (Gper1) is increasingly considered to be playing a role in blood pressure regulation because it acts as a receptor for microbial metabolites (Waghulde et al., 2018). Renal medullary interstitial cell (RMIC) cyclooxygenase-2 (COX-2) deficiency was found to cause salt-sensitive hypertension and papillary damage in response to chronic salt loading (Zhang et al., 2018). The results showed that the blood pressure of WT mice increased significantly after 4 weeks of high-salt diet; the blood pressure increased to a higher degree in ATF4 overexpression mice but did not increase in ATF4 knockdown mice, indicating that ATF4 contributes to the development of hypertension induced by high salt intake in mice. The present study is the first to suggest that ATF4 functions as a blood pressure regulator.

The development of microbiota-targeted emergence and therapy has become a new trend of thought for hypertension. Using high-salt-induced hypertensive mice, Wilck et al. (2017) showed that gut microbiota may serve as a potential target to counteract salt-sensitive conditions. Moreover, Bier et al. (2018) demonstrated a significant association between gut microbiota composition and blood pressure regulation; even the metabolic short-chain fatty acids (SCFAs) may highlight another aspect of the complex interaction between diet, gut, and blood pressure. Additionally, it has been suggested that gut microbiota composition and microbial metabolites are the potential mediators between a single gene associated with blood pressure regulation and hypertension (Faulkner et al., 2018; Waghulde et al., 2018; Yang et al., 2018). In the present study, we found that ATF4 could regulate the gut microbiota composition and the expression of VK2 in hypersensitive mice induced by high-salt diet, suggesting that the mechanisms of ATF4 involved in blood pressure regulation and the vascular endothelial function may be related to gut microbiota and VK2.

Fecal microbiota transplantation has been widely used in several studies to verify microbiota-related mechanisms (Bárcena et al., 2019; Liao et al., 2019; Stebegg et al., 2019; Viennois et al., 2019). In the present study, FMT was performed to verify whether ATF4 involvement in the regulation of blood pressure and endothelial function is associated with the gut microbiota in high-salt-induced hypertensive mice. Our data suggested that the regulation of blood pressure and endothelial function by ATF4 resulted from the gut microbiota composition in salt-sensitive hypertensive mice. Moreover, gut microbiota composition analysis showed that FMT could lower the relative abundance of *norank_ f__Muribaculaceae*, *Lactobacillu*s, *Dubosiella*, *Alloprevotella*, *Allobaculum*, and *[Eubacterium]_xylanophilum_group* and increase the relative abundance of *Lachnoclostridium*, *norank_ f__F082*, *Lachnospiraceae_UCG-006*, and *GCA-900066575* in high-salt-induced hypertensive mice. Furthermore, *norank_ f__Muribaculaceae*, *Lactobacillu*s, *Dubosiella*, *Alloprevotella*, *Allobaculum*, and *[Eubacterium]_xylanophilum_group* may function as probiotics, whereas *Lachnoclostridium*, *norank_ f__F082*, *Lachnospiraceae_UCG-006*, and *GCA-900066575* may function as pathogens. Additionally, *Dubosiella* was considered as a core microbial mediator owing to its high relative abundance (Bai et al., 2019; Fastrès et al., 2020). And the significant difference in abundance among groups was found in current study, which may be associated with ATF4-regulated blood pressure. A previous study indicated that *Dubosiella* was associated with obesity (Bai et al., 2019). A high-fat diet reduced the relative abundance of *Dubosiella*, which may be an important genus for therapeutic effects (Bai et al., 2019). Moreover, the FMT study also indicated that the effects of VK2 might result from the observed changes in the gut microbiota composition associated with ATF4.

Recently, small quantities of VK2 derived from gut microbiota were shown to have a significant impact on health (Bentley and Meganathan, 1982; Bhalerao and Clandinin, 2012; Vos et al., 2012; Ponziani et al., 2017; Haugsgjerd et al., 2020; Ren et al., 2020). A case-control study revealed that an increase in arterial stiffness was associated with an increase in markers of VK2 deficiency (Mansour et al., 2019). Our data confirmed that VK2 was a target metabolite that regulated blood pressure and endothelial function. Moreover, we confirmed the VK2 was absorbed in the serum, as shown in Supplementary Figure 2B. Our data also suggested that VK2 was associated with *Dubosiella*, which was consistent with the previous correlation analysis (Figure 4), indicating that *Dubosiella* may play a role in VK2 metabolism. However, this function needs to be verified through further studies. Our data showed that VK2 significantly decreased ATF4 expression in salt-sensitive hypertensive mice. Therefore, the significant changes in gut microbiota composition and VK2 levels not only confirmed the microbiota-related mechanisms of ATF4 in regulating blood pressure but also suggested that *Dubosiella*-VK2-associated pathways may be the key biomarkers of formative and therapeutic effects in salt-sensitive hypertension. Host genetics and gut microbiota interactions contribute to the pathogenesis of a disease (Chu et al., 2016; Wang et al., 2016). In the present study, we investigated the role of ATF4 in blood pressure regulation in salt-sensitive hypertensive mice. In this study, vascular endothelial function-related factors such as NO, ET-1, ANGI, and VEGF were used to evaluate the vascular endothelial function, and the TEM was used to reflect the structural changes of vascular endothelial cell, which further confirmed the regulation of blood pressure by ATF4. Therefore, our study shows that gut microbiota and VK2 play a role in the participation of ATF4 in hypertension, and these interactions are the possible potential mechanism of ATF4 participating in high-salt diet-induced hypertension. Of course, the detailed mechanism remains to be further studied.

## Conclusion

In summary, ATF4 plays an important role in regulating gut microbiota composition and VK2 expression, thus participating in the development of salt-sensitive hypertension, providing new insights into the association between ATF4 and development of salt-induced hypertension in mice, meanwhile contributing to the development for a new preventive strategy of hypertension.

## Data Availability Statement

The datasets generated in this study are available from the corresponding author upon request. 16S sequencing data have been uploaded to NCBI SRA with accession number PRJNA660089.

## Ethics Statement

The Animal Experiment Protocol listed has been reviewed and approved by the Laboratory Animal Ethics Committee of Jinan University.

## Author Contributions

T-hL and L-gC designed the experiments and analyzed the data. T-hL, W-cT, and Q-eL performed the experiments. T-hL, L-gC, YX, and W-qT wrote the manuscript. All authors read and approved the final manuscript.

## Conflict of Interest

The authors declare that the research was conducted in the absence of any commercial or financial relationships that could be construed as a potential conflict of interest.

## Footnotes

1 http://drive5.com/uparse/

2 http://rdp.cme.msu.edu/

3 www.i-sanger.com

## Abbreviations

ANGI: angiotensin I
ATF4: activating transcription factor 4
ELISA: enzyme-linked immunosorbent assay
ET-1: endothelin-1
IBD: inflammatory bowel disease
NO: nitric oxide
OTUs: operational taxonomic units
SBP: systolic blood pressure
TEM: transmission electronic microscope
VEGF: vascular endothelial growth factor
VK2: vitamin K_2_.
