# Supplementary material for: Gut Microbiota-Related Evidence Provides New Insights Into the Association Between Activating Transcription Factor 4 and Development of Salt-Induced Hypertension in Mice
Source: Front Cell Dev Biol. 2020 Nov 13;8:585995. doi: 10.3389/fcell.2020.585995 (PMC7691383; doi:10.3389/fcell.2020.585995)
Supplement: Supplementary file 1 [file Image_1.pdf]

Supplementary materials

Supplementary figure legends

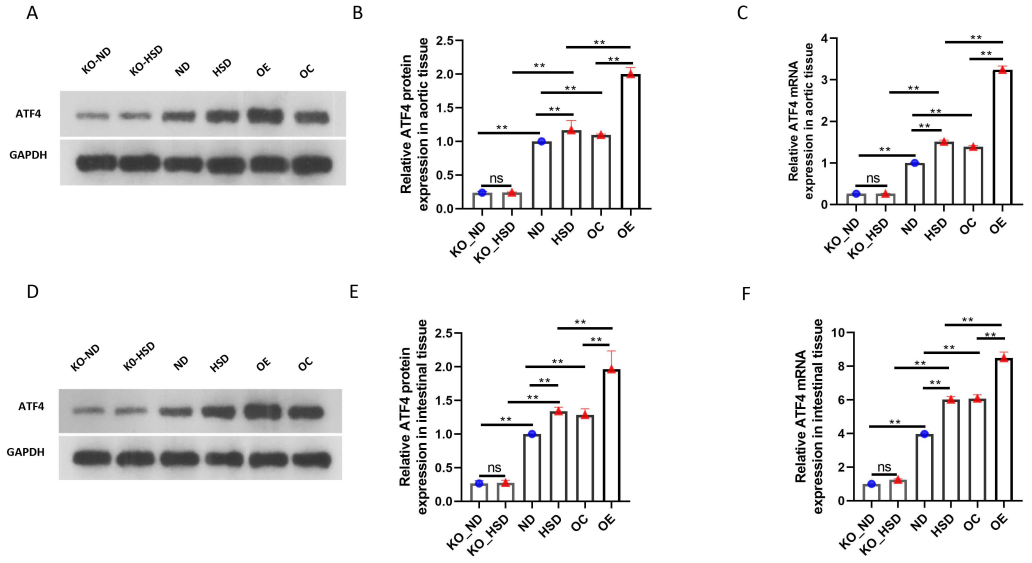

Supplementary figure 1 Relative ATF4 expression. (A) The western blotting of indicated proteins of ATF4 in aortic tissue. (B) The quantification of ATF4 protein levels in aortic tissue. (C) The quantification of ATF4 mRNA levels in aortic tissue. (D) The western blotting of indicated proteins of ATF4 in intestinal tissue. (E) The quantification of ATF4 protein levels in intestinal tissue. (F) The quantification of ATF4 mRNA levels in intestinal tissue. Data are presented as mean ± SD; ns, no significance, *$p < 0.05$ and **$p < 0.01$, n = 3; Statistical comparisons were performed using Student t test or one-way analysis of variance (ANOVA).

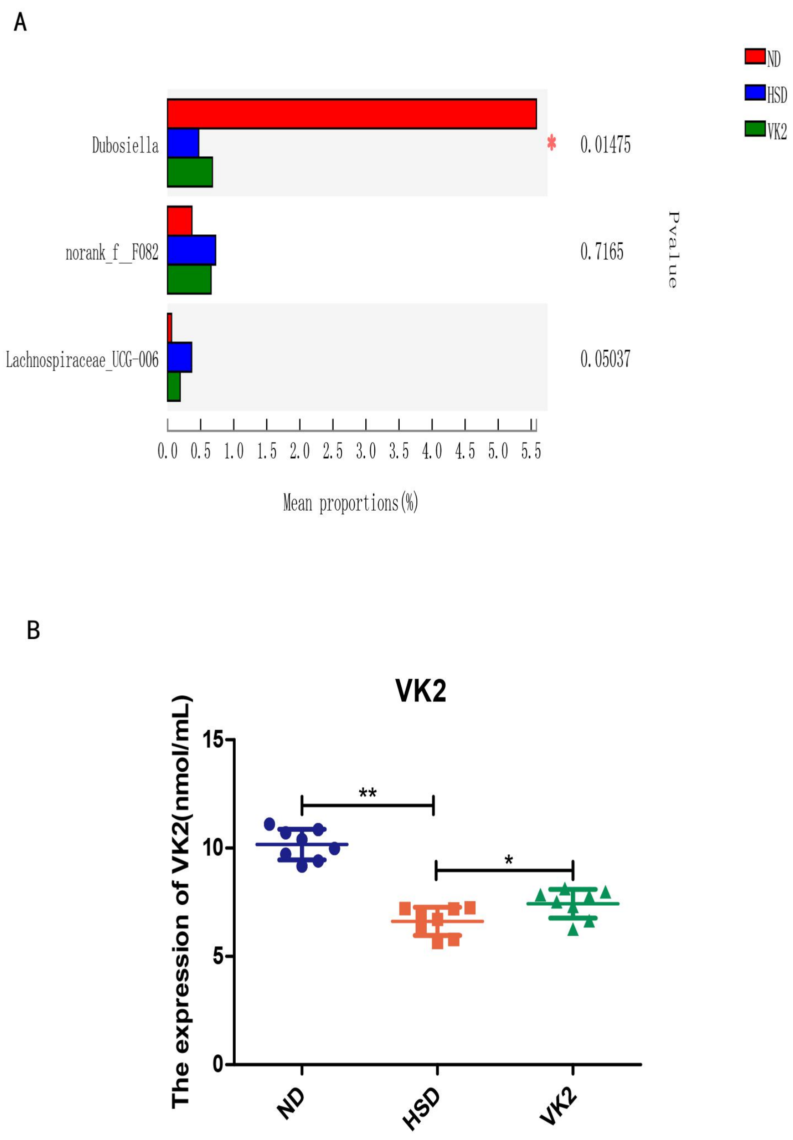

Supplementary figure 2 VK2 affected serum VK2 expression levels and quantities of related microbiota in high-salt-induced hypertensive mice. (A) Related microbiota; *p < 0.05 and **p < 0.01. (B) Expression of VK2. Data are presented as mean ± SD; *p < 0.05 and **p < 0.01, n = 6-8; Statistical comparisons were performed using Student t test or one-way analysis of variance (ANOVA).

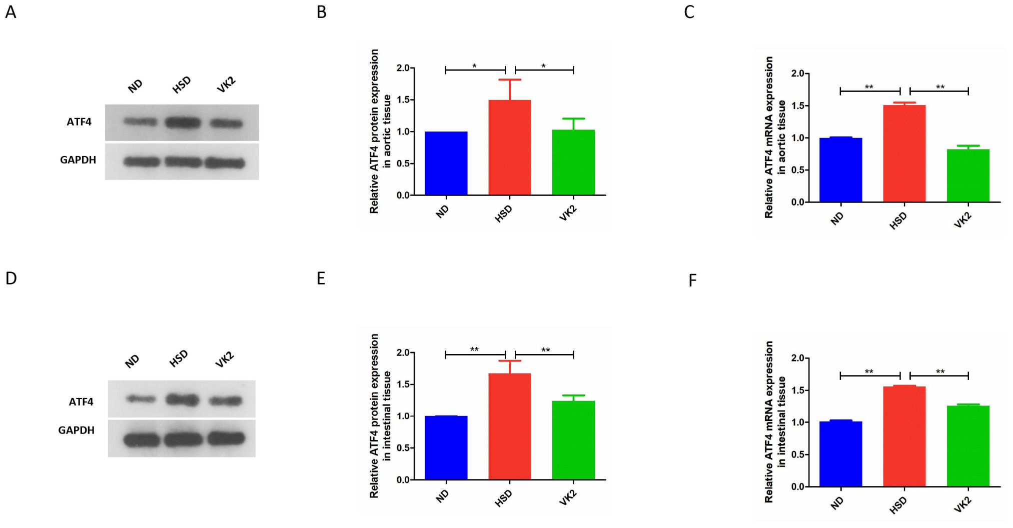

Supplementary figure 3 VK2 decreased ATF4 expression in high-salt-induced hypertensive mice. (A) The western blotting of indicated proteins of ATF4 in aortic tissue. (B) The quantification of ATF4 protein levels in aortic tissue. (C) The quantification of ATF4 mRNA levels in aortic tissue. (D) The western blotting of indicated proteins of ATF4 in intestinal tissue. (E) The quantification of ATF4 protein levels in intestinal tissue. (F) The quantification of ATF4 mRNA levels in intestinal tissue. Data are presented as mean ± SD; *p < 0.05 and **p < 0.01, n = 3; Statistical comparisons were performed using Student t test or one-way analysis of variance (ANOVA).

Supplementary table 1 Primer Information

GENE	5'SEQUENCE	3'SEQUENCE	Size
ATF4	CAAAACAAGACAGCAGCCACTA	CTTCTTCCCCCTTGCCTTAC	186 bp
GAPDH	TGGCCGTGGGGCTGCCCAG	GGAAGGCCATGCCAGTGAGC	107 bp
